# Multiview learning for understanding functional multiomics

**DOI:** 10.1371/journal.pcbi.1007677

**Published:** 2020-04-02

**Authors:** Nam D. Nguyen, Daifeng Wang

**Affiliations:** 1 Department of Computer Science, Stony Brook University, Stony Brook, New York, United States of America; 2 Department of Biostatistics and Medical Informatics, University of Wisconsin-Madison, Madison, Wisconsin, United States of America; 3 Waisman Center, University of Wisconsin-Madison, Madison, Wisconsin, United States of America; University of Pittsburgh, UNITED STATES

## Abstract

The molecular mechanisms and functions in complex biological systems currently remain elusive. Recent high-throughput techniques, such as next-generation sequencing, have generated a wide variety of multiomics datasets that enable the identification of biological functions and mechanisms via multiple facets. However, integrating these large-scale multiomics data and discovering functional insights are, nevertheless, challenging tasks. To address these challenges, machine learning has been broadly applied to analyze multiomics. This review introduces multiview learning—an emerging machine learning field—and envisions its potentially powerful applications to multiomics. In particular, multiview learning is more effective than previous integrative methods for learning data’s heterogeneity and revealing cross-talk patterns. Although it has been applied to various contexts, such as computer vision and speech recognition, multiview learning has not yet been widely applied to biological data—specifically, multiomics data. Therefore, this paper firstly reviews recent multiview learning methods and unifies them in a framework called multiview empirical risk minimization (MV-ERM). We further discuss the potential applications of each method to multiomics, including genomics, transcriptomics, and epigenomics, in an aim to discover the functional and mechanistic interpretations across omics. Secondly, we explore possible applications to different biological systems, including human diseases (e.g., brain disorders and cancers), plants, and single-cell analysis, and discuss both the benefits and caveats of using multiview learning to discover the molecular mechanisms and functions of these systems.

## Introduction

Hierarchical complexity is the nature of all biological phenomena and processes. Although made of physical entities (e.g., atoms), the phenomena and interaction of biological entities such as DNA and proteins—among others—possess emergent properties that cannot be reduced to or explained by physical laws, which have kept biological sciences more descriptive than predictive for a long time. There exists no deterministic law in biology apart from the central dogma, which has actually been questioned and adjusted many times [1, 2]. The flow of genetic information in the central dogma is inherently complex and involves many levels of molecules and interactions (e.g., transcription, translation, alternative splicing, various kinds of regulation mechanisms). To understand a biological phenomenon, we thus need a holistic approach that integrates all the facets and interactions of a biological system as well as collects and analyzes these hierarchical complex data as thoroughly as possible. For this reason, the biological sciences have solely made considerable progress since the era of omics and big data.

High-throughput technologies and next-generation sequencing (NGS) data enable modeling biological systems for understanding underlying complex molecular mechanisms. As correspondents to levels of information flow in central dogma, biological big data are also multileveled and often referred to as multiomics data (i.e., genomics, transcriptomics, epigenomics, proteomics, metabolomics). By combining these “omics,” the complex big biological data can be tackled to disclose relationships between biological entities and identify biomarkers characterizing biological systems. However, a significant challenge involves having access to a set of computational methods powerful enough to shed light on these big data. Accompanied by the strides made in high-throughput biology, machine learning is prospering in biomedical applications, although making sense of multiomics data with traditional machine learning methods nevertheless remains elusive.

The obstacles to doing so are the heterogeneous and implicitly noisy nature of biological data. In fact, omics data are found in many forms, such as sequences (e.g., RNA-Seq, Assay for Transposase-Accessible Chromatin using sequencing [ATAC-Seq]), graphs (e.g., metabolic pathways, regulatory networks), geometric information (e.g., binding site, protein folding), and spatial components (e.g., cell compartment). Biological variables can be continuously or discretely measurable or categorical and may originate from various sources that render them multimodal (rather than Gaussian). These data are often noisy or inconsistent because of the technical problems associated with biological assays, such as background effects and hybridization noise, among others. Furthermore, high-dimensional data (e.g., gene expression profiles with tens of thousands of genes across a limited number of experimental conditions) often suffer from the “curse of dimensionality,” which may lead to overfitting [3].

**Fig 1:**
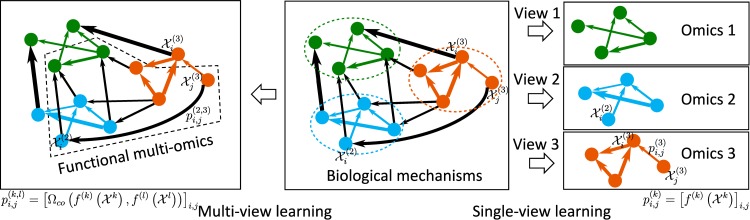
Multiview learning deciphers mechanisms across functional omics. Molecular mechanisms (Center) are resulted from the interactions within and across multiomics, e.g., shown by green, orange, and blue color. The interactions within each omics are illustrated by colored links that matches with the color of that omics; the interactions across different omics are demonstrated by black links. Directed edges represent causal relationships. Edge weights represent relationship strengths. The single-view learning methods (Right) can only learn the within-view interactions separately for each omics via the functions *f*^(*k*)^,*k* = 1,2,3 (e.g., $p_{i,j}^{\left(3\right)}={\left[f^{\left(3\right)}(X^{\left(3\right)})\right]}_{i,j}$). The multiview learning methods (Left) can reveal the cross-talk patterns among various omics, providing complete mechanistic insights on biological functions, e.g., by co-regularization terms Ω_*co*_. These cross-talk patterns are contributed by each facet of learning in either alignment-based methods or factorization-based methods. For example, gene regulatory mechanism can relate to genomics (e.g., regulatory variants), transcriptomics (e.g., gene expression), and proteomics (e.g., TFs). Then Ω_*co*_(*f*^(2)^,*f*^(3)^) represents that variants (e.g., SNPs) break the TFBSs (e.g., $p_{i,j}^{\left(2,3\right)}={\left[\Omega_{co}(f^{\left(2\right)}(X^{\left(2\right)}),f^{\left(3\right)}(X^{\left(3\right)}))\right]}_{i,j}$ as in the figure). Ω_*co*_(*f*^(1)^,*f*^(3)^) represents that variants affect gene expression (e.g., eQTLs). Ω_*co*_(*f*^(1)^,*f*^(2)^) represents that TFs control target gene expression. The multiview learning can thus predict gene regulatory mechanisms across omics on how variants break TFBSs to affect gene expression. eQTL, expression quantitative trait loci; SNP, single-nucleotide polymorphism; TF, transcription factor; TFB, transcription factor binding site.

These challenges are not effectively addressed by traditional machine learning methods; relying on one single data type may lead to either an incomplete understanding of complex processes or overfitting. To address these problems, multiview machine learning offers a solution by integrating different modes or views of data such that learning from this integration leads to greater accuracy and effectiveness. This method is also effective because each mode (or view) is an aspect of the whole complex phenomenon or process that is often compatible and complementary to other modes (or views). Each view can regularize the hypothesis associated with or infer missing data and reduce noise from other data views. Multiview learning has a long history [4] and is used to fuse various data types, such as video, voice, and text. As mutiomics big data is thriving, a comprehensive survey for different methods of multiview learning and their applications to multiomics or biomedical data analysis is necessary, especially for discovering functional omics (Fig 1). For example, a recent paper reviewed the multiview clustering methods with applications to cancer omics [5]. To extend the generality of multiview learning in terms of both modeling and applications, in this review, we formulate multiview learning in a unified mathematical framework called multiview empirical risk minimization (MV-ERM), an extension of empirical risk minimization (ERM) originally introduced by Vapnik [6]. In particular, we firstly introduce the concept of multiview learning, build the 2 formal alignment-based and factorization-based ERM frameworks, and categorize state-of-the-art multiview methods into these 2 categories. Finally, we review some recent biomedical applications of these methods for understanding functional omics and discuss some related problems and conclusions.

### Single-view versus multiview learning

The advancement of high-throughput technologies, which has resulted in tremendous amounts of biological data, has transformed biology from a descriptive science into a predictive science in which machine learning plays an important role. Although biological data are different from visual or speech data, all machine learning algorithms share a common mathematical background that can be described as the ERM principle [6]. In the following sections, we provide the formal descriptions of supervised and unsupervised learning alongside their corresponding ERM estimators.

#### Single-view learning

Biological data are represented by feature vectors, as is the case in other domains, wherein the *i*-th datapoint in a data set $X=[x_{1},\ldots ,x_{n},]\subset X$ is a vector *x*_*i*_ of measured values (e.g., gene or protein expression levels) across different samples (e.g., timepoints, experimental replicates). Each datapoint might be labeled or associated with a particular phenotype *y*_*i*_∈*Y* (i.e., tumor or normal). In a supervised setting, when given an unlabeled datapoint (i.e., a gene expression), we can predict the phenotype (disease or controlled) associated with that datapoint; this prediction is often encoded by function *f*:*X*→*Y*. In an unsupervised setting, we can discover a latent structure from unlabeled data, such as a clustering structure of gene expression profiles in which genes with similar expression levels are grouped together driven by particular molecular functions. In general, the formal definitions of supervised and unsupervised learning are presented next.

#### Supervised learning

**Fig 2:**
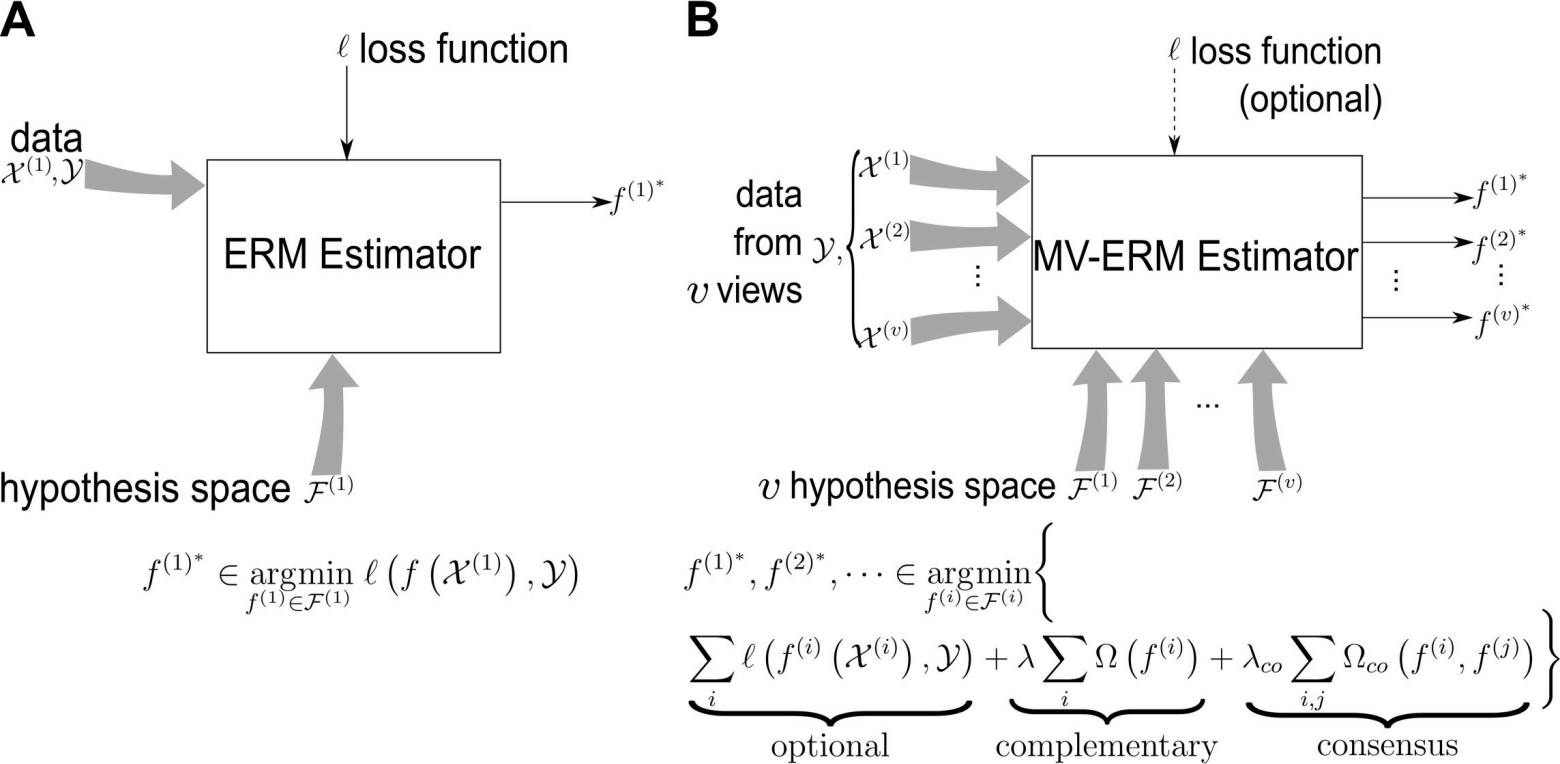
MV-ERM. (A) ERM for single-view learning. It demonstrates a general single-view learning algorithm (based on ERM estimator) that takes one data set $X^{\left(1\right)}$ as input, adopts a hypothesis space $F^{\left(1\right)}$ and a loss function l, and outputs a function $f^{\left(1\right)}\in F^{\left(1\right)}$ that predicts the label associated with any new datapoint *x* as *f*^(1)^(*x*). **(B)** MV-ERM demonstrates a general multiview learning algorithm (based on MV-ERM estimator) that takes *v* datasets $X^{\left(1\right)},\ldots ,X^{\left(v\right)}$ as *v* views, adopts *v* hypothesis spaces $F^{\left(1\right)},\ldots ,F^{\left(v\right)}$ associated with *v* views, and outputs *v* functions $(f^{\left(1\right)},\ldots ,f^{\left(v\right)})\in F^{\left(1\right)}\times \ldots \times F^{\left(v\right)}$ that reveals the interactions within and between each pair of datasets (via the terms Ω_*co*_(*f*^(*i*)^,*f*^(*j*)^). The consensus and complementary principles are implemented by the term Ω(*f*^(i)^) and Ω_*co*_(*f*^(*i*)^,*f*^(*j*)^) respectively. Note that in MV-ERM estimator, the loss function is optional because multiview learning can be unsupervised. ERM, empirical risk minimization; MV-ERM, multiview empirical risk minimization.

In supervised setting, we have *n* labeled examples $S={\left\{\left(x_{i},y_{i}\right)\right\}}_{i=1}^{n}$ where $y_{i}\in Y$ (the label set) and $x_{i}\in X$ (the domain), sampled from an unknown underlying joint distribution over X×Y. The goal is to find a function f:X→Y in a hypothesis space F that predicts the output associated to any new pattern x∈X by *f*(*x*), as measured with respect to a known loss function l(f(x),y). Note that function *f* stands for any transformation, ranging from linear projection to deep neural network and kernel function. For a candidate function f∈F, its empirical risk [6] is

$$R\left(f\right)=\frac{1}{\left|S\right|}\sum_{(x_{i},y_{i})\in S}l\left(f(x_{i}),y_{i}\right)$$ (1)

and the regularizer controlling its smoothness is *Ω*(*f*) where $\Omega :F\to R_{+}$ is a penalty function ($R_{+}$ is the set of nonnegative real numbers). The penalized ERM estimator is

$$f^{*}\in {arg\;min}_{f}R(f)+\lambda \Omega (f)$$ (2)

where *λ* is regularization parameter. For example, support vector machine (SVM) is the combination of hinge loss and $l_{2}$-regularizer, whereas ordinary least squares makes use of squared loss, etc. The basic idea of supervised learning is demonstrated in Fig 2A.

### Unsupervised learning

In unsupervised setting [7], we have *n* unlabeled examples $S=\{x_{i}{\}}_{i=1}^{n}$ where $x_{i}\in X$ sampled from an unknown underlying distribution over X. The goal is to find a latent structure Y (low-dimensional or clustering representation, latent factors, etc.) from X encoded by function f:X→Y in hypothesis space F and decoded by function g:Y→X in hypothesis space G, as measured with respect to a reconstruction error $l\left(\{g,f\},x\right)={\left\|x-g\circ f(x)\right\|}_{2}^{2}$. For a pair of candidate functions {g,f}∈G×F, its empirical risk is

$$R(g,f)=\frac{1}{\left|S\right|}\sum_{x_{i}\in S}l(\{g,f\},x_{i})$$ (3)

The ERM estimator is

$${\{g}^{*},f^{*}\}\in {arg\;min}_{g,f}R(g,f)$$ (4)

Using a reconstruction error, this framework is general enough to encompass a variety of algorithms, such as principal component analysis (PCA), *k*-means, nonnegative Matrix factorization (NMF), autoencoder, etc. In fact, the equivalence of NMF and spectral and *k*-means clustering was investigated [8]. Both *k*-means and PCA methods can be considered as special cases of autoencoders [9]. Several studies have been explored the additional constraints for an autoencoder to perform NMF [10, 11, 12, 13]. Also, NMF has a good interpretability because, for example, it factorizes a gene expression profiles into 2 matrices, one of which describes the structure between genes while the other describes the structure between samples [14]. It also has good performances, especially in single-cell studies [15]. Because of the equivalence of NMF and other unsupervised methods, we represent here the formal settings of NMF as a typical case of unsupervised learning without loss of generality:

Given a data set represented by a nonnegative matrix *X*, NMF decomposes *X* into the production of nonnegative matrices *G* and *F*, i.e., *X*≈*GF*^*T*^. The ERM estimator of NMF can be formulated as follows:

$$\{G^{*},F^{*}\}\in \underset{G,F\geq 0}{\arg\;\min}{\left\|X-GF^{T}\right\|}_{F}^{2}$$ (5)

Note that the objective function (5) takes the matrix form of unsupervised ERM estimator (4) where all *x*_*i*_ from *S* are column vectors of matrix *X*, *f*(*X*) = *F*, and *G* is the matrix representation of the linear operator *g*(⋅).

### Pitfalls of single-view learning

Through the formal definitions of machine learning identified previously, its applications in biological domains can be regarded as abstracting out a representation f(X) of a single data type X, where X can be, for example, a gene expression profile. This representation captures the interactions of elements (i.e., genes) within X and the phenotypic manifestation Y (e.g., cancer) resulting from those interactions. However, to understand complex traits in which the genotype—phenotype interactions manifest over multiple levels of information flows, relying on merely one single omics data type is limited and prohibits our knowledge from uncovering the comprehensive mechanism that underlies complex biological processes. Even with the availability of more than one data type (e.g., $X^{\left(1\right)}$ for gene expression, $X^{\left(2\right)}$ for methylation level), because they originate from different distributions, applying machine learning algorithms on these data independently may solely assemble some pieces of the puzzle of a complex phenomenon; many additional pieces associated with the interactions across different data types remain unknown. Therefore, a learning method that exploits not only information captured in each omic but also that infers from the associations between different omics is needed to understand complex traits. Multiview machine learning is such a method.

### Multiview learning

Many real-life datasets comprise diverse views or modalities; for example, a website can contain both images and texts that refer to the same content. In multimedia applications, for instance, both the speech and lip motions of a character are often simultaneously accessible. These views or modalities are often compatible, which helps the learning model be more robust as well as complementary and thereby reveal further information that cannot be fully uncovered when depending on only one view.

In biology, the need for multiview data is quite trivial; on one hand, we have homogeneous biological data assayed from the same molecular level (e.g., gene or protein expression), yet these homogeneous data may be measured across different conditions, phenotypes, or species. In this context, comparative analysis is important to, for example, find a conserved gene set that functions in the same pathway between 2 different species. On the other hand, multiomics is heterogeneous data in which we have different omics (e.g., genomics, proteomics, epigenomics) assayed from the same tissue or cell. These various omics are encoded by different data views, such as $X^{\left(1\right)}$ for transcriptomic abundance or $X^{\left(2\right)}$ for protein concentration. The goal of multiview learning is to exploit multiple representations of the input data and improve the learning performance. Herein, we set up 2 formal frameworks of multiview learning: one based on the ERM principle of supervised learning (Eq 2) and the other based on the ERM principle of unsupervised learning (Eqs 4 and 5). We also analyze the following 2 characteristics of multiview data that underlie those frameworks: consensus and complementary.

### Consensus and complementary principles

The consensus principle seeks to maximize the agreement among multiple distinctive representations of the data. In short, given an example $x=(x^{\left(1\right)},x^{\left(2\right)})\in X^{\left(1\right)}\times X^{\left(2\right)}$ being seen in 2 views with its label *y*∈*Y*, the goal is to maximize the probability:

$$\max\left(Pr\left[f^{\left(1\right)}(x^{\left(1\right)})-f^{\left(2\right)}(x^{\left(2\right)})=0\right]\right)$$ (6)

The complementary principle demonstrates that in a multiview learning problem, each representation or view may contain information that does not exist in other views. Therefore, combining different views makes the predictor more accurate, or in other words, improves the learning performance [16, 17]. The 2 principles are demonstrated in Fig 2B.

### MV-ERM

In multiview setting, we have *n* labeled examples $S=\{(x_{i},y_{i}{)\}}_{i=1}^{n}$ and *m* unlabeled examples $U=\{x_{i}{\}}_{i=n+1}^{n+m}$, where $y_{i}\in Y$ and each example *x* = (*x*^(1)^,*x*^(2)^,…,*x*^(*v*)^) is seen in *v* views with $x^{\left(i\right)}\in X^{\left(i\right)}$ for *i* = {1,2,…,*v*}. *S* and *U* are both sampled from an unknown underlying joint distribution over $X^{\left(1\right)}\times X^{\left(2\right)}\times \ldots \times X^{\left(v\right)}\times Y$. The goal is to find *v* functions in *v* hypothesis spaces $\{f^{\left(1\right)},\ldots ,f^{\left(v\right)}\}\in F^{\left(1\right)}\times \ldots \times F^{\left(v\right)}$ where $f^{\left(i\right)}:X^{\left(i\right)}\to Y$, predicting the output associated to any new pattern $x\in X^{\left(i\right)}$ by *f*^(*i*)^(*x*), as measured with respect to a known loss function $l^{\left(i\right)}\left(f^{\left(i\right)}(x),y\right)$. For a candidate function $f^{\left(i\right)}\in F^{\left(i\right)}$, its empirical risk is

$$R(f^{(i)})=\frac{1}{\left|S\right|}\sum_{(x_{j}^{\left(i\right)},y_{j})\in S}l^{\left(i\right)}\left(f^{\left(i\right)}(x_{j}^{\left(i\right)}),y_{j}\right)$$ (7)

And the regularizer controlling its smoothness is *Ω*(*f*^(*i*)^), where $\Omega :{⋃}_{i}F^{\left(i\right)}\to R_{+}$ is a penalty function. Also, to impose a penalty on the complexity of each pair (*f*^(*i*)^,*f*^(*j*)^) in a cross-product of 2 hypotheses in order to utilize unlabeled data in different views, we define the co-regularizer

$$\Omega_{co}\left(f^{\left(i\right)},f^{\left(j\right)}\right)=\frac{1}{\left|U\right|}\sum_{x^{\left(i\right)},x^{\left(j\right)}\in U}\Omega_{co}\left(f^{\left(i\right)}(x^{\left(i\right)}),f^{\left(j\right)}(x^{\left(j\right)})\right)$$ (8)

The penalized MV-ERM estimator is

$${\left({f^{\left(i\right)}}^{*}\right)}_{i=1}^{v}\in {arg\;min}_{f^{\left(i\right)}}\{\sum_{i=1}^{v}R\left(f^{\left(i\right)}\right)+\lambda \sum_{i=1}^{v}\Omega \left(f^{\left(i\right)}\right)+\lambda_{co}\sum_{i=1,i<j}^{v}\Omega_{co}\left(f^{\left(i\right)},f^{(j)}\right)\}$$ (9)

In Eq 9, the last term, co-regularizer *Ω*_*co*_(⋅), preserves the consensus principle for multiview learning. Note that if *λ*_*co*_ = 0, this problem reduces to solving 2 independent problems, meaning only the complementary principle is used. In the following, we present 2 frameworks, i.e., alignment-based and factorization-based, for multiview learning that covers most of the recent methods. The basic idea of multiview learning is demonstrated in Fig 2B.

**Alignment-based framework.** In the Eq (9) of MV-ERM estimator, if there is no labeled data, i.e., *n* = 0 (or *S* = ∅), the problem is then

$${\left({f^{\left(i\right)}}^{*}\right)}_{i=1}^{v}\in {arg\;min}_{f^{\left(i\right)}}\{\sum_{i=1}^{v}\Omega \left(f^{\left(i\right)}\right)+\lambda_{co}\sum_{i=1,i<j}^{v}\Omega_{co}\left(f^{\left(i\right)},f^{\left(j\right)}\right)\}$$ (10)

which can be seen as an alignment problem finding a set of embeddings (*f*^(1)^,…,*f*^(*v*)^) that transform the original multiview data into a new common space by identifying an alignment strategy denoted by the co-regularizer Ω_*co*_(⋅). This co-regularizer serves as a pairwise symmetric alignment function across all different views to coordinate the information among them. This multiview framework is based on supervised setting of single-view machine learning in Eq (2) where the loss function is optional, so the learning algorithm will try to uncover the *v* functions not by comparing with a ground truth (i.e., *y*_*i*_) but by comparing to each other (in a pairwise fashion), depicted by the co-regularization term Ω_*co*_(*f*^(*i*)^,*f*^(*j*)^). The co-regularization term can be correlation-based, in which the 2 embeddings *f*^(*i*)^(*x*^(*i*)^),*f*^(*j*)^(*x*^(*j*)^) are maximally correlated, or distance-based, in which the Euclidean distance between the 2 embeddings is minimized. This kind of multiview learning can be regarded as self-supervision in which the *v* learners try to learn from each other’s data.

**Factorization-based framework.** The second framework for multiview learning is based on single-view unsupervised learning (Eq 4), trying to seek a common latent representation for multiple different views. In terms of NMF (Eq 5), given a multiview nonnegative data set consisting of *v* different views as $X^{\left(1\right)},\ldots ,X^{\left(v\right)}$, for each view $X^{\left(i\right)}$, multiview NMF factorizes *X*^(*i*)^≈*G*^(*i*)^*F*^(*i*)*T*^, where *F*^(*i*)^ = *f*^(*i*)^(*X*^(*i*)^), and learns a common latent representation $\tilde{F}$ across all the views via the following MV-ERM estimator:

$${\left(\left\{G^{\left(i\right)}{}^{*},F^{\left(i\right)}{}^{*}\right\},{\tilde{F}}^{*}\right)}_{i=1}^{v}\in \underset{G^{\left(i\right)},F^{\left(i\right)},\tilde{F}\geq 0}{\arg\;\min}\sum_{i=1}^{v}\left\{{\left\|X^{\left(i\right)}-G^{\left(i\right)}F^{\left(i\right)}{}^{T}\right\|}_{F}^{2}+\lambda {\left\|F^{\left(i\right)}-\tilde{F}\right\|}_{F}^{2}\right\}$$ (11)

where *λ* is the regularization parameter, trying to balance the importance of different views and the reconstruction error. The latent representations *F*^(*i*)^ in different views are forced to be close to the consensus one $\tilde{F}$ [18]. In any deep learning architecture, the joint latent representation can be achieved by a joint layer preceded by separated layers corresponding to separated multiple view inputs.

Both 2 aforementioned frameworks can be regarded as representation learning approaches. Whereas in the alignment-based framework, data representations from each pairs of views are forced to be coordinated, the representation of all views in the factorization-based framework are forced to be the same. The consensus principle is demonstrated by the co-regularizer Ω_*co*_(*f*^(*i*)^,*f*^(*j*)^) in the alignment method and by common latent representation (or sometimes called dictionary) $\tilde{F}$ in the factorization method; the complementary principle is demonstrated by the regularizer Ω(*f*^(*i*)^) in alignment methods and by expansion coefficients *G*^(*i*)^ in factorization methods.

### Multiomics interpretation of multiview learning

**Fig 3:**
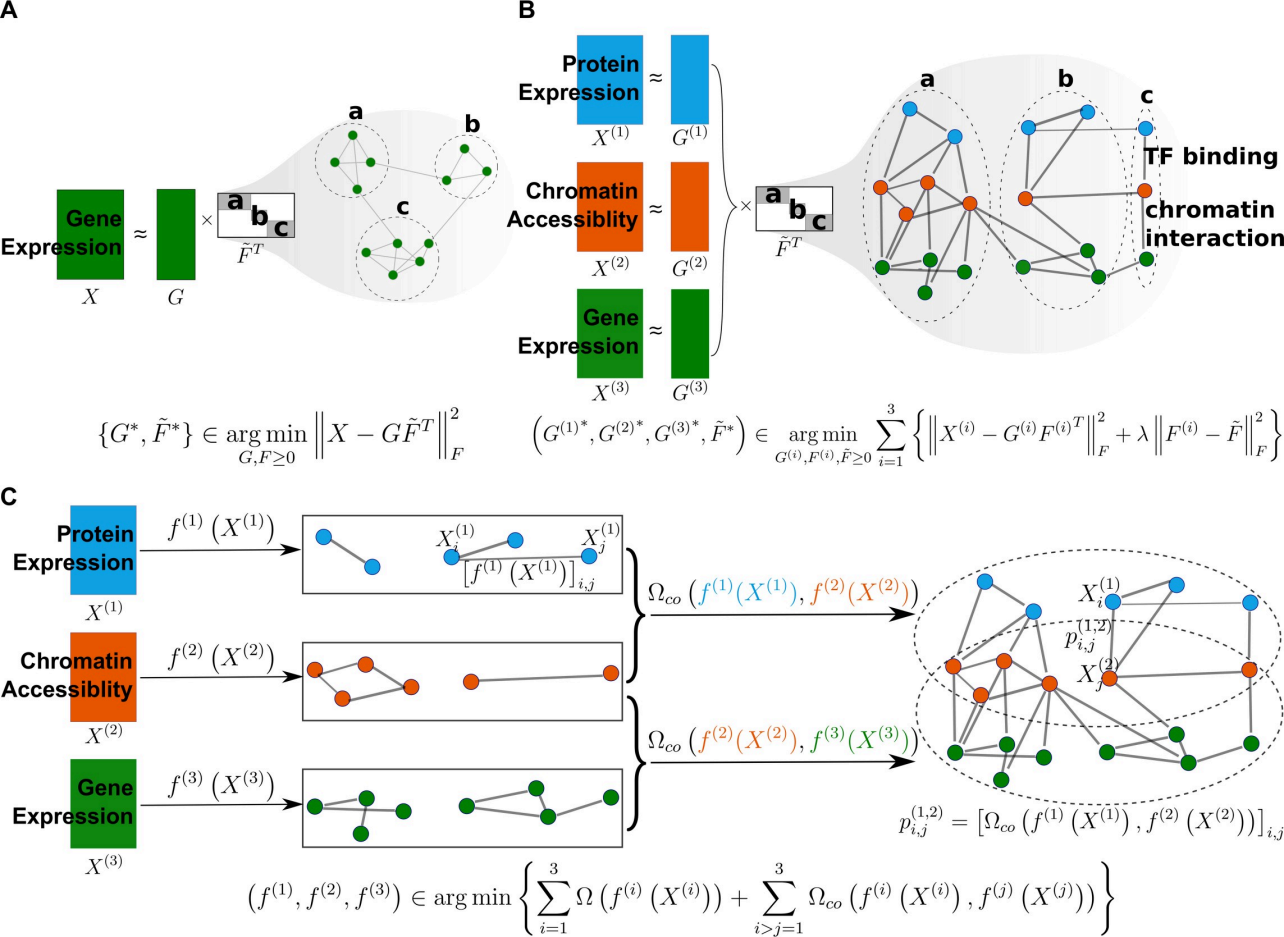
Factorization-based versus alignment-based methods. (A) Factorization-based single-view learning methods. They typically factorize a data matrix *X* from single view (e.g., gene expression matrix of samples by genes) into a product of matrix *G* (coefficient matrix) and matrix $\tilde{F}$ (dictionary matrix or pattern matrix). Because matrix factorization has an intrinsic clustering property [8], the matrix $\tilde{F}$ can represent a clustering structure of the view (i.e., the soft clustering assignments or indicators). For example, $\tilde{F}$ reveals 3 different gene clusters, a, b, and c, as denoted in the figure. (B) Factorization-based multiview learning methods. They factorize different matrices from multiomics, e.g., gene expression *X*^(1)^ (i.e., green matrix), protein expression *X*^(2)^ (i.e., blue matrix), and chromatin accessibility *X*^(3)^ (i.e., orange matrix), into a product of different coefficient matrices *G*^(*k*)^(*k* = 1,2,3) and the common dictionary matrix $\tilde{F}$. This common representation enables revealing of cross-talk patterns among genes, proteins (more precisely, TFs), and regulatory elements (i.e., enhancers); e.g., a TF binds to a region to regulate a gene's expression. (C) Alignment-based multiview learning methods. The 3 input omic matrices are projected via functions *f*^(*k*)^(*k* = 1,2,3) onto spaces where their internal relationships are revealed. These representations of different omics are pairwise coordinated to each other via the term Ω_*co*_. For example, the figure demonstrates the pairwise alignments between *X*^(1)^, *X*^(2)^ and between *X*^(2)^, *X*^(3)^ to reveal cross-talk patterns between TFs and enhancers, and between enhancers and gene expressions. (Alignment between *X*^(1)^ and *X*^(3)^ is not shown for making the figure concise.) TF, transcription factor.

In terms of functional omics, each view of multiview data *X*^(*i*)^ can be a gene expression profile, DNA methylation level, or protein abundance. Multiview learning algorithms applied to these data aim to infer the interactions within each omic, represented by *f*^(*i*)^(*X*^(*i*)^) or *G*^(*i*)^, as well as the interactions across all omics, represented by Ω_*co*_(*f*^(*i*)^(*X*^(*i*)^),*f*^(*j*)^(*X*^(*j*)^)) or $\tilde{F}$. In other words, multiview machine learning attempts to recover a common abstract space wherein the several types of omics data are comparable such that the cross-talk patterns may easily be revealed. For example, in single-view machine learning, gene expression profile clustering is one method of revealing functional modules in which a group of genes collaborate to deliver a biological function. However, the insights of many complex biological processes cannot be understood in terms of these functional modules at the transciptomic level. On the contrary, multiview learning can find a way to represent both gene expression *X*^(*i*)^ and protein expression *X*^(*j*)^ together such that the interactions of genes as well as the interactions between genes and gene products (e.g., proteins) can be captured for a holistic understanding of complex biological phenomena. For example, if gene expression, chromatin accessibility, and protein expression are represented in a common space, they can be simultaneously clustered not only such that a group of genes or a group of proteins that function together can be identified but also—and more importantly—such that the functional linkage between genes, regulatory elements, and proteins can be revealed (e.g., protein *α* binds to region *β* to regulate the expression of gene *γ*). Fig 3 illustrates this example using a factorization method. With a closely related machine learning technique called transfer learning, we can even infer information of an omic level from another omic level. As for homogeneous data across different species, multiview learning can be applied to infer and transfer knowledge from one species to another species [19].

### Multiview learning methods

We categorized all recent state-of-the-art methods of multiview learning into 2 groups according to the frameworks explained in the previous sections: the alignment-based framework, which seeks a pairwise alignment among views, and a factorization-based framework, which seeks a common representation across all views. Each framework contains elements of either consensus principle or complementary principle or both. All the methods described in this review are summarized in Table 1.

**Table 1:** Multiview learning methods.

Method	Multiview Principles	Biological Applications	Data Types	Refs.
Alignment-based				
CCA	Consensus	Cancer, Alzheimer	CSF, MRI, FDG-PET; gene expression, miRNA expression, DNA methylation	[5,20,21]
KCCA/TCCA/SVM-2K	Consensus	Cancer		[22–24]
DCCA	Consensus	Cancer	gene expression, miRNA expression, DNA methylation	[5,25]
DCCAE	Both			[26]
MULPP	Both			[27]
MCCA	Consensus	Cancer	gene expression, miRNA expression, DNA methylation	[5,28]
RGCCA/SGCCA	Consensus	Spinocerebellar Ataxia	metabolomics, lipidomics, magnetic resonance spectroscopy	[29]
PLS	Consensus	Cancer	gene expression, miRNA expression, DNA methylation	[5,30,31]
MvDA/MvDN	Consensus			[32,33]
Manifold Alignment	Both			[34,35]
Manifold Warping	Both			[36]
MATCHER	Both	Single-cell	transcriptomic levels, epigenomic levels	[37]
ManiNetCluster	Both	Plants	gene expression	[19]
MKL	Complementary	Mild cognitive impairment	CSF, APOE genotype, MRI, FDG-PET	[21,38,39]
rMKL-LPP	Complementary	Cancer	gene expression, miRNA expression, DNA methylation	[5,40]
SNF	Complementary	Cancer	gene expression, miRNA expression, DNA methylation	[5,41,42]
NEMO	Complementary	Cancer	gene expression, miRNA expression, DNA methylation	[43]
coupleNMF	Both	Single-cell	gene expression, chromatin accessibility	[44]
Factorization-based				
MultiNMF	Both	Cancer, Alzheimer	gene expression, copy number variation, DNA methylation	[5,18,21,45]
GMvNMF	Both	Cancer	gene expression, miRNA expression, DNA methylation	[46]
Multiview Spectral Clustering	Both	Cancer	gene expression, miRNA expression, DNA methylation	[5,47,48]
Multiview *k*-means	Both	Cancer	gene expression, miRNA expression, DNA methylation	[5,49]
MOFA	Both	Single-cell	RNA expression, DNA methylation, ex vivo drug responses	[50]
iClusterBayes	Both	Cancer	gene expression, miRNA expression, DNA methylation	[5,51]
rMV-spc	Consensus	Cancer	gene expression, PPI network	[52]
Multimodal DNN	Consensus	Mild cognitive impairment	SNP, MRI, PET	[53,54]
Bimodal Deep Autoencode	Consensus			[53]
Multimodal DBM	Consensus			[55]
Multiview CRF	Both			[56]

APOE, apolipoprotein E; CCA, canonical correlation analysis; CRF, conditional random fields; CSF, cerebrospinal fluid; DBM, deep Boltzmann machine; DCCA, deep canonical correlation analysis; DCCAE, deep canonically correlated autoencoder; DNN, deep neural network; GMvNMF, graph regularized multiview nonnegative matrix factorization; MATCHER, manifold alignment to characterize experimental relationships; MCCA, multiway canonical correlation analysis; miRNA, microRNA; MKL, multiple kernel learning; MOFA, multiomics factor analysis; MvDA/MvDN, multiview discriminant analysis/multiview deep network; MULPP, multiview uncorrelated locality preserving projection; NEMO, neighborhood based multi-omics clustering; PET, positron emission tomography; PLS, partial least squares; PPI, protein–protein interaction; RGCCA/SGCCA, regularized generalized canonical correlation analysis/sparse generalized canonical correlation analysis; rMKL-LPP, regularized multiple kernel learning-locality preserving projections; rMV-spc, regularized multiview subspace clustering; SNP, single-nucleotide polymorphism.

#### Alignment-based methods

The consensus principle is realized in alignment-based methods by the co-regularization terms Ω_*co*_(⋅)that coordinate any 2 embeddings $f^{\left(i\right)}(X^{\left(i\right)})$ and $f^{\left(j\right)}(X^{\left(j\right)})$, whereas the complementary principle is realized by the separately regularized feature learning of the different views (i.e., the terms Ω(⋅)).

Canonical correlation analysis (CCA) [20] is one of the first and most popular methods to achieve a consensus between 2 views. Formally, for the 2 datasets *X*^(1)^ and *X*^(2)^, CCA computes 2 linear projections, *F*^(1)^ and *F*^(2)^, such that the cross correlation across 2 views is maximized:

$$\left({F^{\left(1\right)}}^{*},{F^{\left(2\right)}}^{*}\right)\in \underset{\begin{array}{c} F^{\left(1\right)},F^{\left(2\right)}\\ {F^{\left(1\right)}}^{T}X^{\left(1\right)}{X^{\left(1\right)}}^{T}F^{\left(1\right)}=I\\ {F^{\left(2\right)}}^{T}X^{\left(2\right)}{X^{\left(2\right)}}^{T}F^{\left(2\right)}=I \end{array}}{arg\;min}-tr\left({F^{\left(1\right)}}^{T}X^{\left(1\right)}{X^{\left(2\right)}}^{T}F^{\left(2\right)}\right)$$ (12)

Compared with the general form of the alignment-based method in Eq (10), CCA only support the consensus principle, denoted by Ω_*co*_(⋅) = −*tr*(*F*^(1)*T*^*X*^(1)^*X*^(2)*T*^*F*(2)). Note that in Eq (12), the transformation *f*^(*i*)^(⋅) takes the form of a linear projection, represented by matrix *F*^(*i*)^.

Many extensions of CCA support nonlinear embeddings, such as kernel CCA (KCCA) [22], by using the kernel trick to produce a nonlinear version of CCA by implicitly looking for functions *f*^(1)^ and *f*^(2)^ through correspondent kernel functions such that *f*^(1)^(*X*^(1)^) and *f*^(2)^(*X*^(2)^) are maximally correlated. KCCA is also an effective preprocessing step for classification algorithms like the SVM; e.g., SVM-2K [24]. TCCA [23] is a tensor-based extension of CCA capable of handling multiple data views by analyzing the covariance tensor of those views. Deep CCA (DCCA) [25] is a deep learning–based extension of CCA that can be regarded as a parametric alternative to the instance-based method of KCCA to learn correlated nonlinear deep embeddings. Unlike KCCA, DCCA does not require an inner product and does not restrict its hypothesis to a reproducing kernel Hilbert space (RKHS); DCCA also has scalability advantages according to data size. A more recent approach is deep canonically correlated autoencoder (DCCAE) [26], which combines the advantages of both DCCA and deep autoencoder. DCCAE’s architecture is formed by 2 different autoencoders correspondent to 2 views, and although it preserves the autoencoders's reconstruction errors, it also optimizes the canonical correlation between their bottleneck representations. Because of this simultaneous optimization strategy, a trade-off occurs between information learned within each view and information learned across different views. Traditionally, CCA-based approaches implemented only the consensus principle (except for DCCAEs, which have the Ω(⋅)for learning the compact representation of each view). Yet a recent method, multiview uncorrelated locality preserving projection (MULPP) [27], also implemented the complementary principle by preserving the local structures of all the views.

Similar to CCA-based methods that determine the directions of maximum correlation between each view, partial least squares (PLS) finds the directions of maximum covariance. In fact, a correlation can be considered a normalized covariance, and CCA-based methods therefore have close connections with PLS-based methods in several facets [30, 31]. Formally, given a pair of datasets $\left\{X^{\left(1\right)}=\left[x_{1}^{\left(1\right)},\ldots ,x_{n}^{\left(1\right)}\right],X^{\left(2\right)}=\left[x_{1}^{\left(2\right)},\ldots ,x_{n}^{\left(2\right)}\right]\right\}$, the PLS problem can be expressed as:

$$\left({F^{\left(1\right)}}^{*},{F^{\left(2\right)}}^{*}\right)\in \underset{\begin{array}{c} F^{\left(1\right)},F^{\left(2\right)}\\ {F^{\left(1\right)}}^{T}F^{\left(1\right)}={F^{\left(1\right)}}^{T}F^{\left(2\right)}=I \end{array}}{arg\;min}-tr\left({F^{\left(1\right)}}^{T}E[x^{\left(1\right)}{x^{\left(2\right)}}^{T}]F^{\left(2\right)}\right)$$ (13)

We may observe that, similar to CCA, in PLS, the consensus principle is exclusively implemented: $\Omega_{co}(\cdot )=-tr\left({F^{\left(1\right)}}^{T}E[x^{\left(1\right)}{x^{\left(2\right)}}^{T}]F^{\left(2\right)}\right)$. Multiview discriminant analysis (MvDA) [32] can be perceived as the extension of PLS wherein both the between-view and within-view information are considered: $\Omega_{co}(\cdot )=tr\left(\frac{F^{T}SF}{F^{T}DF}\right)$, where $F=[{F^{\left(1\right)}}^{T},\ldots ,{F^{\left(v\right)}}^{T}]$, *F*^*T*^*SF* is the within-class scatter matrix, and *F*^*T*^*DF* is the between-class scatter matrix. Employing a deep architecture, Kan and colleagues [33] also proposed a multiview deep network (MvDN), which aims to achieve a consensus representation of discriminant features across all views. In particular, MvDN consists of 2 subarchitectures—one involving view-specific components *f*^(*i*)^(⋅) for the reduction of view-specific variations and the other involving a common component *g*_*c*_(⋅)for the shared representation across all views. Finally, the loss function of MvDA (i.e., a Fisher-like loss) is applied on the top layer of the network to learn the network’s parameters through backpropagation and gradient descent: $\Omega_{co}(\cdot )=tr\left(\frac{F^{T}SF}{F^{T}DF}\right)$ where *F* = [*g*_*c*_ ∘ *f*^(1)^(*X*_1_),…,*g*_*c*_ ∘ *f*^(*v*)^(*X*_*v*_)].

Whereas most of CCA-based methods solely utilize the consensus principle, manifold alignment can be perceived as an advanced alternative in which both consensus and complementary principles are applied. Manifold alignment is based on the manifold hypothesis, which states that the distribution of real-world high-dimensional data is concentrated near a lower dimensional manifold embedded in the ambient space of the original data. A family of machine learning algorithms (i.e., manifold learning) attempts to capture these data’s manifold structures through nonlinear projections. The idea behind manifold alignment is the aim to capture a low-dimensional common manifold shared by 2 high-dimensional datasets. This aim can be achieved by (1) utilizing 2 nonlinear embeddings (*f*^(1)^(⋅) and *f*^(2)^(⋅)), which transform the 2 original datasets to minimize the distance between them as well as by (2) preserving the geometric structure of each data set. Specifically, given 2 input datasets $\left\{X^{\left(1\right)}=\left[x_{1}^{\left(1\right)},\ldots ,x_{n}^{\left(1\right)}\right],X^{\left(2\right)}=\left[x_{1}^{\left(2\right)},\ldots ,x_{m}^{\left(2\right)}\right]\right\}$, we want to determine the 2 transforms *f*^(1)^(⋅) and *f*^(1)^(⋅) as solutions to this minimization problem:

$$\left(f^{\left(1\right)}{}^{*},f^{\left(2\right)}{}^{*}\right)\in \underset{f^{\left(1\right)},f^{\left(2\right)}}{\arg\;\min}\lambda_{co}\sum_{i,j}\left\|f^{\left(1\right)}(x_{i}^{\left(1\right)})-f^{\left(2\right)}(x_{j}^{\left(2\right)})\right\|W+f^{\left(1\right)}{}^{T}L^{X^{\left(1\right)}}f^{\left(1\right)}+f^{\left(2\right)}{}^{T}L^{X^{\left(2\right)}}f^{\left(2\right)}$$ (14)

where *f*^(1)^ and *f*^(2)^ are functions defined on the respective datasets *X*^(1)^ and *X*^(2)^, and $L^{X^{\left(1\right)}}$ and $L^{X^{\left(2\right)}}$ are the graph Laplacian of *X*^(1)^ and *X*^(2)^, respectively; *W* is the matrix that encodes the correspondences between *X*^(1)^ and *X*^(2)^ such that *W*_*i*,*j*_ = 1 iff $x_{i}^{\left(1\right)}$ corresponds to $x_{j}^{\left(2\right)}$ (e.g., protein $x_{j}^{\left(2\right)}$ is coded by gene $x_{i}^{\left(1\right)}$) [19]. The first term preserves the correspondence (or minimizes the differences) between the 2 views, whereas the second and third terms preserve the local geometric structure of the 2 original datasets by imposing a graph regularization on *f*^(1)^ and *f*^(2)^ [34]. In manifold alignment, Ω_*co*_(⋅) = *λ*_*co*_‖*f*^(1)^(*X*^(1)^)−*f*^(2)^(*X*^(2)^)‖ and $\Omega (\cdot )=f^{\left(i\right)}{}^{T}L^{X^{\left(i\right)}}f^{\left(i\right)}$. Wang and Mahadevan [35] generalized manifold alignment to deal with more than 2 views. To deal with sequence and time series data, Vu and colleagues [36] combined manifold alignment and dynamic time warping [57]. The idea behind graph regularization for multiview learning—as in manifold alignment—is thriving in biomedical applications, for biological networks are pervasive in every level of the analysis.

As the function *f*^(*i*)^ in the alignment-based equation represent any transformation, including the implicit transformation in an RKHS H—because a kernel function $k^{\left(i\right)}(x,x\prime )={\left\langle f^{\left(i\right)}(x),f^{\left(i\right)}(x\prime )\right\rangle }_{H}$—a broad range of multiple kernel learning (MKL) methods [38, 39] can be considered to be alignment-based methods. The basic idea of MKL aims to combine (in a linear or nonlinear way) various kernels that present different notions of similarity from multiple datasets into one additive kernel. In particular, given *v* datasets *X*^(*i*)^ with *v* corresponding kernel matrices *K*^(*i*)^ (for *i* = {1,2,…,*v*}) denoting the similarity over pairs of data points in *X*^(*i*)^, we introduce a new kernel *K*′ = ∑_*i*_*β*_*i*_*K*^(*i*)^, where *β* is a vector of coefficients for each kernel. Because of a kernel’s additivity—a property of RKHS—this new function *K*′ is still a kernel. However, the MKL methods solely support the complementary principle (i.e., Ω(⋅) = *K*^(*i*)^) because we learn merely the appropriate combination of kernels rather than a specific kernel that works most efficiently.

#### Factorization-based methods

In factorization-based methods, the consensus principle is illustrated by the common latent representations $\tilde{F}$ (called the dictionary matrices), whereas the complementary principle is illustrated by the terms *G*^(*i*)^ (called the expansion coefficients matrices).

Many extensions exist for the multiview NMF; for example, multiview clustering via deep matrix factorization [45] employs a seminonnegative matrix factorization to learn the hierarchical semantics of multiview data in a layer-wise fashion. The ERM estimator is:

$${\left({G_{1}^{\left(i\right)}}^{*},\ldots ,{G_{m}^{\left(i\right)}}^{*}{\tilde{F}}^{*},{\alpha^{\left(i\right)}}^{*}\right)}_{i=1}^{v}\in {arg\;min}_{\begin{array}{c} G_{1}^{\left(i\right)},\ldots ,G_{m}^{\left(i\right)},\tilde{F},\alpha^{\left(i\right)}\\ \tilde{F}\geq 0,\sum_{i=1}^{v}\alpha^{\left(i\right)}=1 \end{array}}\sum_{i=1}^{v}{\alpha^{\left(i\right)}}^{\gamma }[{\left\|X^{\left(i\right)}-G_{1}^{\left(i\right)}G_{2}^{\left(i\right)}\ldots G_{m}^{\left(i\right)}\tilde{F}\right\|}_{F}^{2}+\beta tr(\tilde{F}\;L^{\left(i\right)}{\tilde{F}}^{T})]$$ (15)

The first term is the decomposition on all views through *m* layers in which the representations on the last layer are forced to be the same $\tilde{F}$. The learning procedure depends on 2 parameters—*α^(i)^*, which controls the weight for the *i*th view, and *γ*, which controls the distribution of weights such that the important views have high weights. The second term, including *L*^(*i*)^, the graph Laplacian of the *k*-nearest neighbor graph constructed from data view *i*, maintains the geometric structure of each view. The nonnegativity constraint on the hidden representation $\tilde{F}$ makes the model easy to interpret.

As spectral clustering has been proven to be equivalent to NMF [8], multiview spectral clustering is also related to multiview NMF. The idea behind multiview spectral clustering is the aim to achieve a common eigenvector matrix from different views. In particular, the method regularizes all distinct eigenvector matrices towards a consensus matrix by solving the following optimization problem [47]:

$${\left({F^{\left(i\right)}}^{*},{\tilde{F}}^{*}\right)}_{i=1}^{v}\in \underset{\begin{array}{c} F^{\left(i\right)},\tilde{F}\\ {F^{\left(i\right)}}^{T}F^{\left(i\right)}={\tilde{F}}^{T}\tilde{F}=I \end{array}}{arg\;min}-\left[\sum_{i=1}^{v}{F^{\left(i\right)}}^{T}L^{\left(i\right)}F^{\left(i\right)}+\lambda \sum_{i=1}^{v}tr(F^{\left(i\right)}{F^{\left(i\right)}}^{T}\tilde{F}{\tilde{F}}^{T})\right]$$ (16)

Another way to establish the common eigenvector matrix is presented by Cai and colleagues [48], whose optimization problem is formulated as

$${\left({F^{\left(i\right)}}^{*},{\tilde{F}}^{*}\right)}_{i=1}^{v}\in \underset{\begin{array}{c} F^{\left(i\right)},\tilde{F}\\ \tilde{F}\geq 0,{\tilde{F}}^{T}\tilde{F}=I \end{array}}{arg\;min}-\left(\sum_{i=1}^{v}{F^{\left(i\right)}}^{T}L^{\left(i\right)}F^{\left(i\right)}+\lambda \sum_{i=1}^{v}tr[{\left(\tilde{F}-F^{\left(i\right)}\right)}^{T}(\tilde{F}-F^{\left(i\right)})]\right)$$ (17)

where $\tilde{F}\geq 0$ makes $\tilde{F}$ become the final clustering indicator matrix. The main difference between the 2 methods is that the first uses $tr\left(F^{\left(i\right)}F^{\left(i\right)}{}^{T}\tilde{F}{\tilde{F}}^{T}\right)$, and the second adopts $tr\left[{\left(\tilde{F}-F^{\left(i\right)}\right)}^{T}\left(\tilde{F}-F^{\left(i\right)}\right)\right]$ as 2 different terms that measure the lack of consensus between views that must be minimized.

As shown by Ding and colleagues [8], *k*-means clustering can also be formulated as an NMF problem by using an indicator matrix $\tilde{F}$. To deal with large-scale multiview data, Cai and colleagues [49] proposed a multiview *k*-means clustering method that adopts a common indicator matrix across different views. The optimization problem is formulated as follows:

$$\left(G^{\left(i\right)}{}^{*},\alpha^{\left(i\right)}{}^{*},{\tilde{F}}^{*}\right)\underset{\begin{array}{c} G^{\left(i\right)},\alpha^{\left(i\right)},\tilde{F}\\ {\tilde{F}}_{j,k}\in \{0,1\},\sum_{k}{\tilde{F}}_{j,k}=1,\\ \sum_{i=1}^{v}\alpha^{\left(i\right)}=1 \end{array}}{\arg\;\min}\sum_{i=1}^{v}\alpha^{\left(i\right)}{}^{\gamma }{\left\|X^{\left(i\right)}{}^{T}-\tilde{F}G^{\left(i\right)}{}^{T}\right\|}_{F}^{2}$$ (18)

The learning procedure depends on 2 parameters—*α*^(*i*)^, which controls the weight for the *v*-th view, and *γ*, which controls the distribution of weights—such that the important views acquire significant weight during multiview clustering.

As a multiview deep learning extension for autoencoder model, Ngiam and colleagues [53] introduce a bimodal deep autoencoder to extract the shared representation of the bottleneck layer, which is the concatenation of 2 views' code. This fusion forces the compact representations of 2 views to be comparable. Many different network architectures implement the similar idea of a shared top (or bottleneck) layer of various networks associated with different views [58, 59, 60, 61]. Multimodal deep Boltzmann machine [55] is a similar method derived from a probabilistic graphical model approach. In these deep learning methods, the shared layer serves as the consensus principle, whereas layers that belong to different networks serve as the diversity principle. Currently, most of these multiview deep learning methods are merely applied for multimedia data (i.e., sounds and visions), not for biomedical data yet.

Another multiview extension for deep probability model is multiview conditional random fields (multiview CRF) [56], which is used to label sequential data. To implement the consensus principle, the authors used a joint representation for features extracted from different neural networks and then minimize the distance between the 2 views. To implement the complementary principle, they integrated features of multiple views into the framework of CRF. Variational dependent multioutput Gaussian process [62] is also a multiview method for sequential data modeling, which utilizes the Gaussian process.

## Applications

### Cancers

Cancer is a complex disease whose phenotypic manifestation might be related to many different levels of molecular signatures, such as gene expression and DNA methylation. In other words, cancer types and subtypes can be defined based on, for example, both genetic mutations and epigenetic landscapes. Therefore, any causal analysis based on solely one aspect or single omics will be a causal reductionism that might lead to insufficient results. Multiomics approaches in oncology research is thriving [63], and many applications of such approaches have been recently pursued.

Rappoport and Shamir [5] performed an extensive review and benchmark that compare 9 multiview methods on 10 cancer types using cancer datasets from The Cancer Genome Atlas (TCGA) spanning 3 omics—that is, gene expression, microRNA (miRNA) expression, and DNA methylation. Among the 9 algorithms chosen, LRAcluster [64], *k*-means, and spectral clustering are clustering methods that performs on the concatenation of various omics into a single matrix, which is a method often referred to in the literature as early integration [16]. The other 7 methods are similarity network fusion (SNF), regularized multiple kernel learning-locality preserving projections (rMKL-LPP), multiway canonical correlation analysis (MCCA), multiview NMF (MultiNMF), iClusterBayes, and PINS. These methods are chosen to reflect their categorization which, as stated therein, is overlapped; nevertheless, these practically available tools are widely used. The *k*-means, spectral clustering, and multiNMF methods are mentioned in the previous section. iClusterBayes [51] is also a factorization method belonging to the subspace clustering family that shares an objective function similar to that of NMF and *k*-means. MCCA [28] is a generalization of CCA wherein pairwise correlations between embeddings are maximally summed up. The rMKL-LPP method [40] is a case of MKL specifically developed for multiomics data. SNF [41, 42] is a graph-based approach that aims to fuse different graphs that represent various omics and may also be regarded as an MKL method on graph structure. The PINS method [65], belonging to the approach sometimes called late integration [16], is the averaging of all clustering results from different omics. In their paper, Rappoport and Shamir [5] demonstrate that rMKL-LPP performs most efficiently in terms of clinical enrichment, whereas MCCA and MultiNMF perform most efficiently with respect to survival. It is worth noting that rMKL-LPP is a specialized multiomic method, whereas MCCA and MultiNMF are general multiview learning methods.

Rappoport and Shamir [43] also developed a new method for multiomics clustering, neighborhood-based multi-omics clustering (NEMO), which can be applied to partial datasets in which patients' omic data are missing without performing data imputation. The authors also compared NEMO with the benchmark on a previous study [5] and demonstrated an improvement of their method on partial data. The idea behind NEMO is similar to those behind SNF and rMKL-LPP; all 3 methods are MKL approaches. Firstly, the similarity matrix of each omic is built based on a radial basis function kernel. Secondly, theses matrices are integrated into one average relative similarity matrix. Finally, spectral clustering is applied on this unified matrix wherein a modified eigenmap method is employed. The ability of NEMO to handle a partial data set is based on a local neighborhood approach. Experiements revealed that NEMO is faster and simpler than existing multiomics clustering algorithms.

Multiview NMF is also used for the selection of common codifferential genes [46]. In this paper, the authors implemented a graph-regularized version of multiview NMF (GMvNMF) to encode the data manifold of genomic data. Manifold regularization for multiview learning was first introduced in the form of manifold alignment. This kind of geometric information embedding can be applied to any multiview machine learning method, including GMvNMF. This method's validity was tested in 4 cancer multiomics data from TCGA, and each of these cancer types comprises 3 omics (i.e., gene expression, copy number variation, and DNA methylation). GMvNMF was demonstrated to perform more efficiently than other NMF methods, including plain multiview NMF.

Also using graph regularization, Zhang and Ma [52] proposed a regularized multiview subspace clustering (rMV-spc) method to discover common co-expressed modules. These modules serve as biomarkers across various cancer stages that might lead to the revealing of mechanisms that underlie the development of cancers. The graph regularization employed therein is the protein—protein interaction (PPI) network; their optimization procedure is based on interior point algorithm. They performed their method on breast cancer data from TCGA and reached a more favorable result compared with that of an artificial network benchmark. Yet while claiming the method's extensibility to heterogeneous multiomics data, in this study, the authors exclusively included gene expression data. Although the PPI network was also used, it is regarded as prior information in the form of regularization rather than a different source of data view.

Yu and colleagues [66] proposed a method for simultaneous clustering of multiview cancer data using a multiview spectral clustering method. However, their computational method substantially differs from other spectral clustering approaches in that, rather than calculating eigenvectors, the optimization procedure therein involves of a line-search algorithm on Stiefel manifold. In this gradient descent method, the gradient calculated from a Euclidean space in each iteration is projected onto an embedded matrix manifold. The authors applied this method to both simulation and real data, which also originate from TCGA. In both cases, the method performed favorably. In the real data set composed of gene expression, miRNA expression, and DNA methylation across 12 cancer subtypes, their method identified more clusters that are enriched by gene ontology and KEGG pathways, which could be used to explain the different mechanisms for each subtype of cancer development.

### Brain diseases

The nature of mental disorders and neurodegenerative diseases, of which Alzheimer’s disease (AD) is the most common one, remains a puzzle. Although many psychiatry diagnoses are currently based on neuroimaging and, hence, multiview learning from different types of neuroimages (e.g., MRI, fMRI, positron emission tomography [PET], computerized tomography [CT]), a study of AD [67, 68] suggests that memory impairment and dementia are the results of nonlinear interactions involving multiple brain cell types (e.g., neurons, microglia), pathogenic forms of *τ* proteins and amyloid-*β* as the brain ages. Whereas these interactions are preserved in a healthy brain, they are dysfunctional in an unhealthy brain, thereby leading to mild cognitive impairment (MCI) and giving rise to AD. Also, each cell type is affected by unhealthy aging at multiple levels, such as transcriptomic, epigenomic, proteomic, metabolomic, and lipidomic. Therefore, a holistic approach that combines these omic data on blood, cerebrospinal fluid (CSF), brain samples, and also neuroimages as phenotypic traits is essential for revealing the complex mechanism underlying the disease. The combination of omics studies and medical imaging (sometimes called radiogenomics) advances our understanding of AD and neurodegenerative disorders in general at multiple levels through the identification of biomarkers for diagnosis and through association studies that reveal the interaction mechanism among genetic and phenotypic data.

Xu and colleagues [69] developed a Bayesian multiview learning method for association studies and diagnosis of AD through 2 data views: genetic variations (i.e., single-nucleotide polymorphism [SNP]) (discrete ordinal data) and MRI features (continuous data). By using sparse linear projections to factorize common latent features, the method aims to (1) simultaneously discover the interactions between genetic variations and MRI features and (2) select biomarkers associated with the disease. The authors also incorporated the linkage disequilibrium as a prior knowledge for the SNP data.

As also a radiogenomics approach, Zhou and colleagues [54] utilized both MRI and PET images as well as SNP to identify AD’s prodromal status—MCI—and to classify MCI subjects into 2 groups of either progressive MCI (pMCI) or stable MCI (sMCI); these groups are categorized based on who will develop AD and who will remain stable. For diagnostic predicting and for dealing with the heterogeneity and high-dimension, low-sample-size problem of the multiview data, the authors developed a deep multiview network that slowly fuses 3 different datasets into a common representation after a stage-wise training using the “maximum number of available samples”; specifically, the architecture makes use of a “three-stage deep feature learning and fusion framework.” In the first stage, latent representations of each view (MRI, PET, SNP) are learned separately, whereas in the second stage, the joint pairwise representations are learned by using the features in the first stage. In the third stage, diagnostic labels are learned by integrating all features from the second stage. The analysis is made on the AD neuroimaging initiative (ADNI) data set and achieves a favorable performance. The multiview deep learning approach taken therein can be regarded as a slow fusion architecture [70]. Another approach of discriminating MCI subgroups is presented by Young and colleagues [71], wherein the authors made use of Gaussian process as an MKL method to integrate volumetric MRI, fluorodeoxyglucose positron emission tomography (FDG-PET), CSF, and apolipoprotein E (APOE) genotype for a binary classification. This combination of neuroimaging, genomic, and metabolomic data delivered an accuracy of 74%, higher than any results achieved using a single modality. The combination of structural MRI, FDG-PET, and CSF are also used in an experimental study [21] in which 3 different multiview learning methods—namely CCA, MKL, and matrix factorization—are compared on the ADNI data.

For late-onset AD (LOAD), Mukherjee and colleagues [72] proposed a general multiview framework for feature learning of 27 previously known driver genes of LOAD, which may then be used to identify other potential driver genes. The authors also proposed a ranking method for these genes by aggregating the predictions associated with each feature set with genome-wide association study (GWAS) statistics. While claiming the framework's generality for any data modalities, the authors demonstrated the analysis via 3 modes of data: differentially expressed genes between AD cases and controls, global gene co-expression network features, and 42 tissue-specific co-expression modules. These transcriptomic data are collected from postmortem brain tissue across 3 different studies that are assumed to possess independently predictive information. To tackle sparsely labeled data (of only 27 known genes), they developed a multiview classification based on a co-training scheme. For feature learning, they indicated that topological features (e.g., node degree) are more predictive than are differential expression features; for ranking, they identified previously known and also potentially new LOAD driver genes that are significantly enriched for both SNPs and pathways associated with AD.

Another degenerative genetic disease is spinocerebellar ataxia (SCA), which is responsible for severe movement disorders. This complex disease, which possesses more than 40 genetically different types, must also be studied from an integrative approach that makes use of omics data, neuroimaging data, and clinical data, among others. Garali and colleagues [29] analyzed 4 subtypes of SCA, which are SCA1, SCA2, SCA3—the 3 most common subtypes—and SCA7, by employing component-based methods known as regularized generalized CCA (RGCCA) and sparse generalized CCA (SGCCA). These methods generalize CCA to analyze data sets structured in blocks, each of which represents a unique view of data. This kind of analysis aims to reveal information between and within blocks. Because SCA is characterized by the volume of a brain region called the pons, the authors performed RGCCA and SGCCA as block-based multimodal biomarkers approaches to discover the relationships between the pons volume, metabolomics, lipidomics, and metabolic imaging resulting from magnetic resonance spectroscopy.

### Single-cell omics

Cellular populations are heterogeneous in nature. Although cells in a particular tissue are of the same type (e.g., neuron, muscle), they are nevertheless varied in terms of their states (e.g., mitotic, migratory) and behaviors according to the transcriptomic, proteomic, and other measurement levels in a spatiotemporal pattern. Research based on bulk sample of cells from a specific tissue undermines these variations across cells so that the emerged single-cell technologies flourish and thereby enable the exploration of cellular heterogeneity in complex diseases and stem cell differentiation. Single-cell multiomics provide diverse views for each individual cell (e.g., genomic, epigenomic, transcriptomic) that suggest how these different molecular levels interact to result in a phenotypic heterogeneity of cellular types, states, and fates (e.g., the effects of DNA methylation in the cell population on gene expression [73, 74, 75]). Integrating these multiomics remains a challenge, especially when sparseness and high dimensionality are the 2 pervasive characteristics of single-cell multiomics data [76, 77]. Sparseness is caused by dropout events wherein the gene expression is very high in some cells but very low or almost zero in other cells due to the stochastic nature of gene expression at the resolution of single-cell. The mixing of these false zeros with true zeros of nonexpressed genes makes the analysis difficult. Thus, to impute missing values from an omic, we need information from other omics. Also, the high dimensionality caused by the large number of genes in each cell would make any approach for discrimination between cells be very hard because, in this high-dimensional space, the distance between cells is indistinguishable.

Few imputation methods in single-cell analysis make use of multiview learning from multiomics data. Lin and colleagues [78] developed an ensemble regression imputation method that combines the self-imputation from a single omics (e.g., miRNA) as well as cross-imputation from other correlated omics (e.g., mRNA, DNA methylation). When comparing 5 other single-view imputation methods, the method presented therein was demonstrated to be advanced and efficient in terms of imputation accuracy and the recovery of mRNA-miRNA interactions. Multiomics factor analysis (MOFA) [50] is another multiomics integrative method that can efficiently identify outlier samples and accurately impute missing values. The method learns a set of hidden factors responsible for biological and technical variability from different omics and clearly identifies the consensus information shared across multiple omics as well as the diversity of specific information that belongs to individual omics. The inferred factors enable the identification of sample subgroups, data imputation, and sample outliers. When applied to a data set of chronic lymphocytic leukaemia on 200 patient samples, including somatic mutations, RNA expression, DNA methylation, and ex vivo drug responses, MOFA identified many sources of disease heterogeneity, such as immunoglobulin heavy-chain variable region status, trisomy of chromosome 12, and response to oxidative stress. When applied to single-cell multiomics data, MOFA identified coordinated transcriptional and epigenetic changes along cell differentiation. The ensemble method used by Lin and colleagues [78] can be regarded as an alignment-based method because it makes use of correlations between omics, whereas MOFA is a factorization-based method that attempts to reveal common latent factors.

The high dimensionality of single-cell multiomics requires any integration method to consider dimension reduction one of its goals. This requirement is naturally implemented in factorization-based methods because they attempt to reveal a common latent structure that often resides in a low-dimensional space. The desired result may also be accomplished if the embeddings *f*^(*i*)^ in alignment-based methods transform the original data to a linear or nonlinear manifold. Welch and colleagues [37] developed MATCHER to perform manifold alignment between transcriptomic and epigenomic levels from different cells. The method firstly uses a Gaussian process latent variable model to obtain pseudotime values for every cell by independently clustering them in every omics and secondly aligns the quantiles of the pseudotime distribution and those of a uniform distribution to make them directly comparable. As far as we understand, very few computational methods in bioinformatics—especially in multiomic integration—utilize the method of manifold alignment even though manifold structure is a suitable representation for gene regulatory networks because it preserves the locality of regulons [79]. Similar to MATCHER, ManiNetCluster [19] is another method for multiview learning that attempts to identify conserved or specific gene modules across species via manifold alignment. Although the data used in their studies are merely transcriptomic profiled from bulk samples, the method is general sufficient to apply to single-cell multiomics data to identify cell types, cell states, and even the functional linkage between various omics. It is worth noting that not all NMF methods used in single-cell multiomic integration are factorization-based approaches. Duren and colleagues [44] developed a method called coupleNMF to cluster cells using both gene expressions (scRNA-seq) and chromatin accessibility (scATAC-seq). This method does not recover a common dictionary matrix that captures the consensus across different views, as is the case in other multiview NMF methods; rather, it identifies the association between genes and regulatory elements and is thus a co-regularized method.

### Plants

Multiomics and machine learning may be applied in plant science, especially to understand the mechanisms of photosynthesis and hydrogen metabolism that are valuable for biofuel research. *Chlamydomonas reinhardtii* is a microalgae often used as a premier reference organism to study biohydrogen production because of its high hydrogenase activity [80]. For example, a study applied both transcriptomic and proteomic levels to reveal a majority of the algal genomes being differentially expressed over the course of the light condition and the timing of specific genes being determined by their biological functions [81]. Another study implemented of genomics, transcriptomic, proteomics, and metabolomics to identify critical genes in hydrogen metabolism [80]. The combination of transcriptomic, proteomic, metabolite, and lipid profiling was also used to investigate the the regulation of photosynthetic process during nitrogen deprivation in *C*. *reinhardtii* [82]. However, these studies exclusively applied basic statistical techniques in their analysis and as such, lacked a systematic method for integrating and inferring from different types of omics.

As far as we understand, ManiNetCluster [19] is the only multiview learning method that has been used in plant science. In general settings, the method takes 2 different data sets as inputs, transforms them into a common latent subspace where they can be aligned with each other, then simultaneously clusters the aligned network for the discovery of conserved modules and functional linkages between 2 data types. In their study [19], a gene expression profile of *C*. *reinhardtii* between light and dark conditions was employed, and the 2 conditions were treated as 2 views of a multiview data set. ManiNetCluster was subsequently applied on these 2 inputs, which led to the discovery of conserved modules in which a group of genes retain their functions during both the daytime and nighttime. Some critical genes that serve as functional linkages to bridge and regulate daytime and nighttime functions were specifically identified.

## Summary and discussions

Multiview learning has a long history [4], and many literature reviews have been produced on this topic, including the following: Li and colleagues [83] focus on multiview representation learning methods; Zhao and colleagues [84], Sun [85, 86], Sun and colleagues [87] focus on some theoretical aspects—that is, generalization bounds—of some old paradigms of multiview learning (e.g., co-training); one of the first reviews discussing extensively on the consensus and complementary principles of multiview learning is made by Xu and colleagues [16]; Chao and colleagues [88] focus on and categorize multiview clustering methods into generative and discriminative methods; and Baltrušaitis and colleagues [89] conducted a comprehensive survey that categorizes multiview learning methods into 5 technical challenges—representation, translation, alignment, fusion, and co-learning. Most methods surveyed by Baltrušaitis and colleagues [89] are general or specialized for multimedia applications. The applications of multiview learning in biomedical data are just recently investigated [90, 91], and there are also surveys investigating the methods to integrate heterogeneous biological and multiomics data [92, 93, 94, 91]. However, they did not discuss the underlying machine learning principles (e.g., ERM) for multiview learning and how to use these principles for modeling multiomics data and revealing functional omics.

Different from these reviews, we focused on the basic principle underlying all machine learning algorithms (i.e., ERM) and built the alignment-based and factorization-based frameworks for multiview learning based on that principle. We can categorize nearly all the multiview learning methods into those 2 framework by demonstrating which components of their objective functions are responsible for the consensus or the complementary principles of multiview learning. These 2 forms of MV-ERM may also be employed in a future theoretical analysis to derive the generalization bounds of a learning algorithm. We have demonstrated that, with the general settings, our multiview learning framework may be either supervised or unsupervised. In fact, the alignment-based methods are based on supervised settings of single-view machine learning, whereas factorization-based methods are based on the reconstruction error of single-view unsupervised learning. The alignment-based methods are always performed in pairwise fashion and therefore not scalable, as is the factorization-based method; however, in parallel to data integration, alignment-based methods may be applied for an association or comparative analysis.

Current machine learning methods based on ERM have some potential pitfalls, e.g., for understanding causal relationships between variables. When minimizing empirical error, the learning algorithm tries to absorb all the association relationships (e.g., correlation) found in the data. To tackle this association-versus-causation dilemma, Arjovsky and colleagues [95] proposed a theoretical framework, invariant risk minimization (IRM), to learn causations by inferring invariances across conditions (e.g., different omics in biological context). This opens up the possibility to generalize IRM to multiview settings (i.e., multiview IRM) for learning the directed links among variables across omics, implying potential causal relationships.

There are also a few caveats in multiview learning applications, especially in terms of time and space complexities. First, given that its input is typically multiomic data, multiview learning is computationally costly and demands high data memory usage. For example, alignment-based methods proceed in a pairwise fashion, which probably results in a 2-fold increase of data memory. Moreover, factorization-based methods can result in a higher degree of polynomial space complexity in that they simultaneously process all available datasets. Second, a number of hyperparameters may be used to define a multiview learning model [96]. Tuning various hyperparameters is still challenging. In particular, searching an optimal set of hyperparameters is likely computationally intensive. For example, DCCA [25], a multiview deep learning method, has to simultaneously optimize 2 deep neural networks, creating additional computational burden in training.

Many topics were not covered in this paper. To identify and categorize various kinds of multiview learning methods, we exclusively focused on the algorithms' objective functions but did not discuss the details of their optimization procedures. In fact, many learning methods share similar objective functions but nevertheless differ from optimization methods. Most of the NMF-based methods are based on an alternating optimization technique, whereas spectral methods (e.g., spectral clustering) are based on solving a generalized eigenproblem. Spectral clustering can also be solved by an optimization procedure on a matrix manifold, such as Stiefel manifold [66]. Most deep learning approaches are solved by backpropagation and stochastic gradient descent methods, whereas many other solvers are based on a convex relaxation [64, 97, 98, 76]. There are additional topics that are closely related to multiview learning, such as domain adaptation and transfer learning, that we were unable to dig into in this paper despite their biological application; in our outlook, we find the inferring of the information from an omic to another omic more promising. Also, biological interpretability is still a challenge for machine learning applications. To address this, previous work embedded biological knowledge to the machine learning model for underlying mechanisms; e.g., interpretable deep neural network modeling [99, 15]. Thus, how to make multiview learning interpretable will be an interesting topic in near future. For biological applications, we herein focused on the cutting edge of cancer, neurodegenerative diseases, and single-cell multiomics. The many other applications we were unable to cover include epigenomic variation, gene regulation, and computational pharmacology (e.g., drug repositioning, patient subtyping), among others. These applications can be identified in other surveys, such as [76]. As for benchmark datasets, in addition to cancers [5], we also summarized additional multiomic benchmark datasets for additional contexts (S1 Table).

We also acknowledge that many multiview learning models (especially deep learning models), although popular in domains such as computer vision and speech recognition, are not currently applied in the biological domain. To move forward, we may take example of other domains. For example, a multiview clustering based on deep matrix factorization [45] learns features via a hierarchical model with multiple layers. Each layer learns a feature representing specific data attributes; e.g., a portrait photo has attributes of pose, facial expression, and facial identity. Clustering photos based on this multiview learning model enables the simultaneous identification of the features and the relationships among photo attributes. Similarly, the idea of identifying hierarchical features in this model can be potentially applied to single-cell data for understanding cell-type-specific gene expression and identity. For example, we can input single-cell gene expression matrices (genes by cells) [100] and learn the features representing (1) cell identity (e.g., cell type) and (2) cell activity (e.g., gene expression) as well as the feature relationships (e.g., cell type interactions).

Another research that has a great influence in healthcare in recent years is the study of the microbiome community inhabiting the host or an environmental niche. For example, metagenomics studies of the gut microbiome have shown the changes of community structures under the changes of diet [101]. However, metagenomics constitute merely another one view for the whole understanding of complex phenotypic traits; to understand the whole microbial traits, we need to integrate metagenomics with other omics and meta-omics (e.g., metatranscriptomics, metaproteomics) in a multiview framework. Among various multiomics integration, combining metabolomics with metagenomics is a promising way to understanding functions and interactions between microbial community and the host [102].

In short, we have provided the formal framework for categorizing current multiview learning methods; it can also serve as a guideline for developing many new methods. We demonstrated that the biological applications of these methods are thriving and promising, especially in the fields of brain diseases (e.g., neurodegenerative and neurodevelopmental diseases) and single-cell analysis because of the growing use of multiomics data. Biological problems always involve of many diverse facets, and multiview learning is an efficient strategy for tackling those problems. We expect that, through this review, additional applications and issues in multiview learning research shall emerge and benefit the community.

## Supporting information

S1 Table Multiomics benchmark datasets. (XLSX)
(XLSX)
